# Molecular and In Silico Analysis of the *CHEK2* Gene in Individuals with High Risk of Cancer Predisposition from Türkiye

**DOI:** 10.3390/cancers16223876

**Published:** 2024-11-20

**Authors:** Ozkan Ozdemir, Brittany L. Bychkovsky, Busra Unal, Gizem Onder, Ufuk Amanvermez, Eylul Aydin, Berk Ergun, Ilayda Sahin, Merve Gokbayrak, Cansu Ugurtas, Merve Nur Koroglu, Berfin Cakir, Irem Kalay, Naci Cine, Ugur Ozbek, Huma Q. Rana, Ozden Hatirnaz Ng, Nihat Bugra Agaoglu

**Affiliations:** 1 Department of Medical Biology, School of Medicine, Acibadem University, 34752 Istanbul, Türkiye; ozkan.ozdemir@acibadem.edu.tr; 2 Rare Diseases and Orphan Drugs Application and Research Center (ACURARE), Acibadem University, 34752 Istanbul, Türkiye; gizem.senturk@live.acibadem.edu.tr (G.O.); ufukamanvermez@gmail.com (U.A.); eylul.aydin@live.acibadem.edu.tr (E.A.); ilayda.sahin@live.acibadem.edu.tr (I.S.); ugur.ozbek@ibg.edu.tr (U.O.); 3 Division of Cancer Genetics and Prevention, Dana-Farber Cancer Institute, Boston, MA 02215, USA; 4 Department of Medical Oncology, Dana-Farber Cancer Institute, Boston, MA 02215, USA; 5 Harvard Medical School, Boston, MA 02115, USA; 6 Department of Medical Genetics, Division of Cancer Genetics, Umraniye Training and Research Hospital, 34764 Istanbul, Türkiye; busraunalbav@gmail.com (B.U.); dr.iremgenetic@gmail.com (I.K.); 7 Department of Molecular Biology and Biochemistry, Institute of Health Sciences, Acibadem University, 34752 Istanbul, Türkiye; 8 Department of Genome Studies, Institute of Health Sciences, Acibadem University, 34752 Istanbul, Türkiye; 9 Geniva Informatics and Health Services Incorporated Company, 34752 Istanbul, Türkiye; berk.ergun@live.com; 10 Department of Medical Biotechnology, Institute of Health Sciences, Acibadem University, 34752 Istanbul, Türkiye; 11 Department of Medical Genetics, School of Medicine, Kocaeli University, 41001 Izmit, Türkiye; merve_ertan09@hotmail.com (M.G.); cansu.ugurtas@gmail.com (C.U.); nacicine@yahoo.com (N.C.); 12 Department of Biostatistics and Bioinformatics, Health Sciences Institute, Acibadem University, 34752 Istanbul, Türkiye; mervenurelif@gmail.com; 13 Department of Genetics and Bioengineering, Istanbul Bilgi University, 34060 Istanbul, Türkiye; berfin.cakir@bilgiedu.net; 14 Izmir Biomedicine and Genome Center (IBG), 35340 Izmir, Türkiye; 15 IKF—Institut für Klinische Krebsforschung GmbH, 60488 Frankfurt, Germany

**Keywords:** *checkpoint kinase 2* (*CHEK2*), cancer predisposition, variant interpretation, variant of uncertain significance (VUS), statistically significant in silico predictor (SSIP)

## Abstract

**Background and Objectives:** *Checkpoint kinase 2* (*CHEK2*) is a tumor suppressor gene involved in DNA repair and cell cycle regulation. Pathogenic or likely pathogenic (P/LP) variants in *CHEK2* are associated with increased cancer risk. Conversely, recent large cohort studies have identified certain variants that, despite being classified as P/LP by in silico analysis, are considered low risk. Thus, the genotype–phenotype correlations of *CHEK2* require a better understanding. In this study, we aimed to characterize germline *CHEK2* variants from a group of individuals who applied to cancer genetic clinics in the Marmara Region of Türkiye. We also aimed to assess the phenotypic impacts of these variants by using a new score of statistically significant in silico predictors (SSIPs). **Methods:** We analyzed 1707 individuals with high risk cancer predisposition, focusing on germline *CHEK2* variants, using SSIP scores and population-specific data. **Results:** *CHEK2* variants appeared in approximately 8% of cases. The SSIP scores indicated that the missense mutation, p.Arg117Gly, significantly impairs DNA repair. Almost half of the variants had higher allele frequencies than the variants listed in the Genome Aggregation Database (gnomAD), and three variants had significantly higher frequencies compared to the variants listed on the Turkish Variome database (p.Thr476Met, p.Arg137Gln, c.592+3A>T), emphasizing the importance of population-specific data. **Conclusions:** This comprehensive analysis of *CHEK2* variants in the Turkish population provides crucial insights for cancer geneticists and oncologists. Our findings will help to enhance the evaluation and management of cancer predisposition associated with *CHEK2* in Türkiye and other regions that have significant Turkish populations.

## Simple Summary

The *CHEK2* gene plays a crucial role in DNA repair mechanisms. In this study, we analyzed *CHEK2* variants in a group of people from Türkiye who had a cancer diagnosis or family history of cancer and investigated how *CHEK2* variations increase cancer risk. We found that 8% of these cases had a variant in *CHEK2*. We used a new scoring method to predict how these changes might affect the cancer risk, which helped to advance previous assessments. Our findings highlight the importance of considering population-specific data when estimating cancer risk and suggest that some variants might add more or less risk than previously thought. This research provides valuable insights for physicians and geneticists working with cancer predisposition both in Türkiye and across the world.

## 1. Introduction

The *checkpoint kinase 2* (*CHEK2*) gene, which encodes the CHK2 protein, is activated in response to DNA damage and is involved in DNA repair [1]. Due to its important role in vital cellular processes, genetic mutations in *CHEK2* that influence CHK2 protein function affect cancer risk. Germline pathogenic and likely pathogenic (P/LPs) variants in *CHEK2* increase an individual’s risk of breast, colorectal, kidney, prostate, and thyroid cancer [2,3,4,5]. One of the most common pathogenic variants in *CHEK2* is c.1100del. This variant is found five times more frequently in BRCA-negative breast cancer cases than in healthy controls [6].

The majority of *CHEK2* variants are missense mutations, and currently, many missense variants in *CHEK2* are classified as variants of uncertain significance (VUS). Missense pathogenic or likely pathogenic (P/LP) variants in *CHEK2* seem to confer a similar cancer risk to the loss-of-function variant 1100delC. Several ongoing efforts are currently underway to classify VUSs as P/LPs or benign/likely benign (B/LB) variants. To achieve this, there is a need to understand the variants’ impacts on protein function. In a recent publication, the American College of Medical Genetics and Genomics (ACMG) reported that *CHEK2* specific clinical practice should be used in the management of *CHEK2*-associated cancer risks [7]. Moreover, recent studies have revealed that three common *CHEK2* variants, p.Ile157Thr, p.Ser428Phe, and p.Thr476Met, carry a distinctly lower risk of cancer compared to other *CHEK2* P/LPs [8]. Current National Comprehensive Cancer Network (NCCN) guidelines do not distinguish clinical management between P/LPs in *CHEK2* and the three common low-risk variants (p.Ile157Thr, p.Ser428Phe, and p.Thr476Met).

Today, according to 28 evidence attributions provided by the ACMG/AMP (Association for Medical Pathology), variants are classified into five categories (from benign to pathogenic) [9]. These evidence attributions are divided into 16 pathogenic and 12 benign criteria. Depending on the weight of evidence, the pathogenic criteria are divided into “very strong” (PVS1), “strong” (PS1–4), “moderate” (PM1–6), and “supporting” (PP1–5); the benign criteria are divided into “stand-alone” (BA1), “strong” (BS1–4), and “supporting” (BP1–7). The evidence attributions are also grouped by type, based on population data, functional data, segregation data, de novo data, allelic data, data from other databases, and computational data obtained from in silico prediction tools.

Many in silico pathogenicity predictors (ISPPs) have been developed over the last decade to evaluate a variant’s effect on protein function. One challenge with ISPPs is that ISPP scores can provide contradictory results even for variants with known clinical significance [10]. Most ISPP prediction algorithms use parameters such as evolutionary conservation, changes in the 3D structure and electrochemical properties of the protein, population allele frequencies, etc. as well as meta-scores obtained by powerful machine learning or deep learning algorithms that have such information as their training dataset. In this context, by their very nature, ISPPs inherit the biases contained in the existing data from training data sets in their predictions. The accuracy and efficiency of each ISPP for evaluating genomic variations varies depending on the gene and genomic loci. Therefore, according to the ACMG/AMP 2015 guidelines, “multiple lines of computational evidence” should be evaluated within the scope of supporting evidence (guideline PP3). To overcome this issue, we statistically compared the ranked scores of *CHEK2* variants known to be P/LP, B/LB, and VUS to determine the most accurate ISPPs for *CHEK2*.

In addition to guideline PP3, the ACMG/AMP 2015 guidelines include PS4, which stresses the importance of population data in establishing the rarity of a variant in specific contexts. PS4 supports a pathogenic interpretation when the frequency of a variant is substantially higher in affected individuals compared to controls [9]. While current interpretations of *CHEK2* variants rely primarily on the allele frequencies observed in Western populations, the available dataset is already constrained, particularly in regions like Türkiye, where population studies are limited.

This study aims to delineate the spectrum of *CHEK2* variants among individuals with possible cancer predisposition risk or with hereditary cancer backgrounds in Türkiye. Focusing on those with either a personal history of cancer or familial cancer predisposition, we aimed to assess the utility of ISPPs for variant interpretation within the Turkish population. Our approach involved comparing allele frequencies from our study cohort to reference data from the Genome Aggregation Database (gnomAD) and the Turkish Variome database, followed by a reassessment of the pathogenic potential of *CHEK2* VUSs. In addition, we implemented our novel statistical algorithm, leveraging in silico pathogenicity predictors to generate a weighted ISPP meta score [11], which facilitated the precise classification of *CHEK2* variants into B/LB, VUS, or P/LP categories.

## 2. Materials and Methods

### 2.1. Study Population

A total of 1707 individuals who applied to the Medical Genetics departments of Umraniye Training and Research Hospital (*n* = 1320), Kocaeli University, School of Medicine (*n* = 335), and Acibadem University, School of Medicine (*n* = 52) between 2018 and 2021 were retrospectively analyzed for germline *CHEK2* pathogenic and likely pathogenic (PVs) or variant of unknown significance (VUS) variants. The cases with a positive family history, the cases with a first degree affected family member, the cases who were diagnosed earlier than expected age, the cases who showed bilateral involvement and the cases who showed high toxicity were included in the study cohort. The family members of the index cases were assessed by Sanger sequencing and were not included in the study group.

### 2.2. DNA Extraction and Next-Generation Sequencing

The genomic DNA was purified from the peripheral blood using a QIAamp DNA Mini QIAcube Kit (Qiagen (Hilden, Germany)). The samples were studied by a panel of cancer geneticists, and the prepared libraries were entered into a NextSeq500 (Illumina (San Diego, CA, USA)) instrument with paired end-reads (2 × 150 bp). The raw data that were obtained after the sequencing reaction were analyzed according to the diagnostic workflow of each center. The detailed protocols are provided in the Supplementary Materials and Methods section.

### 2.3. Variant Reinterpretation

The allele counts of *CHEK2* variants between the study cohort, the gnomAD database [12], and the Turkish Variome database were compared statistically. We extracted allele counts and performed Fisher’s exact tests for each variant to assess the significance of the differences in allele frequencies between the groups. Specifically, we constructed 2 × 2 contingency tables for comparisons between the study cohort and the gnomAD database and between the study cohort and the Turkish Variome database. The *p*-values obtained from the Fisher’s exact tests were then adjusted for multiple comparisons using the Bonferroni method to control for the false discovery rate. Variants with missing data in any group were noted as “NA” for non-calculable *p*-values. The results, including the raw and adjusted *p*-values, were compiled and saved in a new tabulated text file for further interpretation. Fisher’s exact test *p* values of <0.05 and Bonferroni-adjusted *p*-values of <0.1 were considered statistically significant.

### 2.4. In Silico Studies

We developed a statistical approach to evaluate the accuracy of various in silico pathogenicity predictors (ISPPs) in classifying *CHEK2* missense variants based on their clinical significance. Initially, all *CHEK2* missense variants were extracted from the dbNSFP (v4.3a) dataset [13]. Variants were pre-filtered based on ClinVar [14] submissions, specifically those meeting the criteria of at least two gold stars (indicating criteria provided, multiple submitters, and no conflicts). These variants were categorized into three main groups: Pathogenic (P/LP), Benign (B/LB), and Unknown (VUS). Pathogenic and likely pathogenic variants were assigned to the Pathogenic group, benign and likely benign variants to the Benign group, and variants of uncertain significance to the Unknown group. Additionally, variants reported in Simple ClinVar [15] were included regardless of submission criteria. In total, 526 missense variants (seven pathogenic, five benign, 514 unknown) of *CHEK2* were utilized for downstream analysis.

The Kruskal–Wallis test, a non-parametric method for analyzing variance, was used to statistically compare the ISPP scores across the three variant groups. Out of 46 ISPPs in the dbNSFP (v4.3a) database, 44 were analyzed (excluding LINSIGHT and CADD v1.9 due to limited compatibility). Each ISPP was ranked by score, facilitating a standardized comparison across variants. Significant differences between variant groups were identified for 17 ISPPs, including ClinPred, MetaRNN, M-Cap, and BayesDeladdAF, among others (*p* < 0.05).

To provide a comprehensive pathogenicity assessment, we developed a combined score for each variant by averaging the ranked ISPP values, integrating Bonferroni-corrected *p*-values to weight ISPPs according to their predictive power for *CHEK2*. The final pathogenicity score was calculated by summing the weighted ISPP scores for each variant and scaling the results between 0 and 1 for consistent comparison. This approach enabled the classification of *CHEK2* missense variants, providing insights into their potential clinical relevance and facilitating more accurate risk assessment.

The splice site variation c.592+3A>T was analyzed using SpliceAI and Pangolin, which provided a comprehensive evaluation of the variant’s potential impact on splicing mechanisms.

### 2.5. Protein Stability Prediction and Structural Conservation Analyses

The protein data bank (PDB) file of the CHK2 protein (Uniprot: O96017) was downloaded from Alphafold: Protein Structure Database (https://alphafold.ebi.ac.uk/, accessed on 1 May 2023). The model’s confidence for regions of interest in the downloaded PDB file was assessed, and each was rated either “very high” or “confident”. Following evaluation of the model, it was uploaded to DynaMut2 to calculate the ΔΔG scores (Figure S1). We chose two low-level likely pathogenic variants, p.Ile157Thr (rs17879961) and p.Ser428Phe (rs137853011), a frequent pathogenic variant, p.Arg117Gly (rs28909982), and the most common variant in our cohort, p.Thr476Met (rs142763740), for the analysis of protein stability changes.

The variant positions may be structurally conserved in low-risk *CHEK2* variants (p.Ile157Thr, p.Ser428Phe, and p.Thr476Met), although evolutionary conservation is not observed in their sequences. Therefore, we further analyzed CHK2 missense variants both sequentially and structurally. In this context, five different PDB files from different species (human, mouse, rat, zebrafish, and yeast) were downloaded from Alphafold and uploaded to DaliServer (http://ekhidna2.biocenter.helsinki.fi/dali, accessed on 1 May 2023) [16] to perform the structural alignment analysis [17]. Lastly, we used the WebLogo2 website (http://weblogo.threeplusone.com/, accessed on 1 May 2023) [18] to create sequence logos for better visualization of the results.

## 3. Results

### 3.1. Clinical Findings

In our study cohort, 131 individuals were positive for *CHEK2* P/LP, a low-risk variant, or a VUS. Among these, 126 cases were female (96.2%), and five were male. The mean age of diagnosis was 45 (range: 19–70). Ninety-nine (75.6%) cases had been diagnosed with breast carcinoma, and nine patients had bilateral disease. In addition, there were eight cases of colorectal cancer, three cases of multiple primary cancers, and nine cases diagnosed with other cancer types (cervical, Ewing sarcoma, ovarian, stomach, and thyroid cancers). More than 60% of the cases had a positive family history, and almost 10% had been tested due to familial risk (Table 1).

### 3.2. CHEK2 Variations Detected in the Cohort

**Figure 1:**
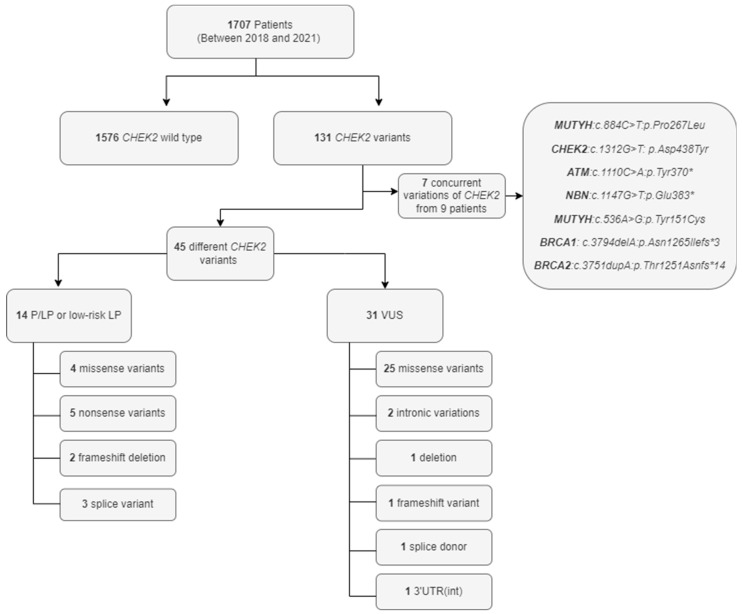
Diagram of the study cohort and the classification of identified *CHEK2* variants. This diagram provides an overview of the study cohort of 1707 patients screened for *CHEK2* gene variants between 2018 and 2021. The flowchart breaks down the classification of patients and the types of *CHEK2* variants identified during the study. The concurrent variations in genes other than *CHEK2* are also emphasized as part of the broader genetic landscape identified in some patients. *: nonsense variation creating a stop codon.

**Figure 2:**
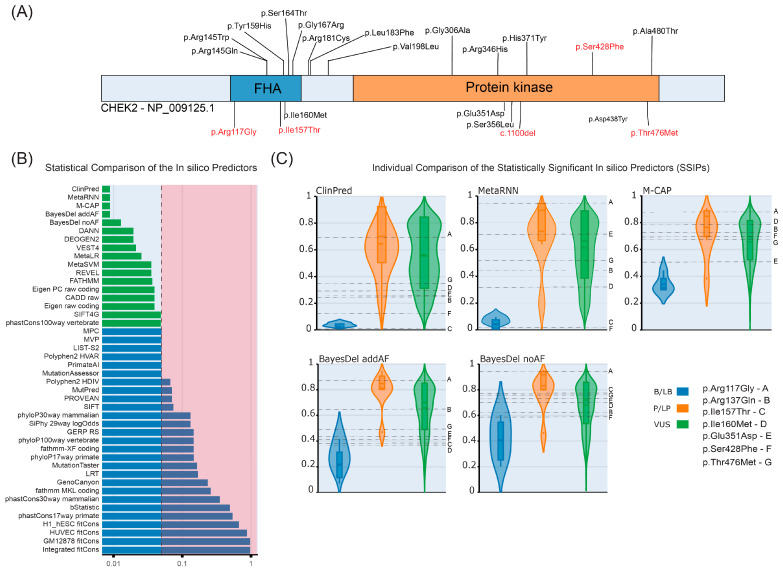
In silico characterization of *CHEK2* variants. (**A**) Mutational landscape of *CHEK2*. All missense and indel variants and their positions in the patient cohort are given in the figure. Red-colored points indicate conflicting VUSs. (**B**) Statistical comparison of the in silico prediction tools for the *CHEK2* gene. The blue bars represent the insignificant in silico predictors. The known benign/likely benign (B/LB), pathogenic/likely pathogenic (P/LP), and VUS variants from the Clinvar and gnomAD databases were compared via Kruskal–Wallis tests, and *p*-values were adjusted according to the Bonferroni method. Green points represent statistically significant in silico predictors (SSIP) with Bonferroni-adjusted *p*-values of <0.05. (**C**) Individual representation of the first five SSIP ranked score comparisons.

A total of 45 different *CHEK2* variations were detected (14 P/LP or low-risk variants and 31 VUSs, Table 2 and Figure 1) among 131 cases. The cohort had 29 missense, five nonsense, four splice donor/acceptor, three frameshift, three intronic, and one in-frame deletion variatios. In addition, nine cases had seven concurrent variations of *CHEK2* with other cancer predisposition genes (Table S1). Six of these cases were diagnosed with breast cancer, one with ovarian cancer and two individuals were studied due to familial risk. p.Thr476Met (18.39.1%) was the most common variation in our cohort. The p.Ile157Thr variant was observed in eight cases (17.4%), whereas c.1100del was observed in two cases (0.01%). Variant p.Ser428Phe was not observed in any of the cases. All variant allele fractions were around 50%, and there were two homozygous cases; one was a PV, and the other was a VUS. The localization of the most common PVs and the PVs determined in this study are illustrated in Figure 2A.

We also compared the allele frequencies (AF) in our cohort with those in the gnomAD database. The AF of 27 variants were significantly higher in our cohort than in the gnomAD data (Table 3). Furthermore, we compared the AF in the cancer cohort with the AF of available variants (*n* = 24, Table 4) among the Turkish Variome data [19]. The AF of three variants (p.Thr476Met, p.Arg137Gln, and c.592+3A>T) were significantly higher in our cancer cohort than in the Turkish Variome data (*p*-values: 0.04, 0.03, and 0.003, respectively) and the p.Ile160Met variation showed a limited *p*-value (*p* = 0.055). When we checked the allele counts, we applied the PS4 evidence only to the c.592+3A>T VUS variant due to its significantly increased allele count in the study cohort (*p*-value = 0.004). In addition, we analyzed the variant using the SpliceAI and Pangolin predictors (https://spliceailookup.broadinstitute.org/#variant=chr22-28724974-T-A&hg=38&distance=500&mask=0&ra=0, accessed on 1 May 2023). Accordingly, the c.592+3A>T VUS variant was predicted to have 0.90 probability of donor-loss at position +3, leading to potential alternative splicing events; thus, we reclassified the variant as LP.

The low-risk LP, p.Thr476Met, showed higher significance in the cancer group than in non-cancerous cases, whereas the p.Ile157Thr variation had similar allele frequency to that detected in the non-cancerous cases.

### 3.3. In Silico Analysis

We evaluated the functional effects of selected *CHEK2* variants (p.Arg117Gly, p.Arg137Gln, p.Ile157Thr, p.Ile160Met, p.Glu351Asp, p.Ser428Phe, and p.Thr476Met) through ISPPs, allele frequencies, and simulations for protein stability changes in CHK2. Among the 44 ISPPs included in the analysis (Table S2), the ClinPred, MetaRNN, M-Cap, BayesDel addAF, BayesDel noAF, DANN, DEOGEN2, VEST4, MetaLR, MetaSVM, REVEL, FATHMM, Eigen PC raw coding, CADD, Eigen raw coding, SIFT4G, and phastCons100way vertebrate tools found statistical significance (*p* < 0.05) for the *CHEK2* gene (SSIPs for *CHEK2*: Figure 2B, Table S4). A combined score was determined for each variant of interest in this study by averaging the ranked values of the SSIPs obtained from the dbNSFP database (version 4.3A) (Figure 2B,C, Figure S2 and Table S3).

Our evaluation obtained the highest SSIP combined score (0.88) from p.Arg117Gly. Moreover, the protein stability test showed a destabilizing effect of −1.84 kcal/mol for the same variant. p.Arg117Gly was not present in our cohort or in the Turkish Variome database. Considering the relatively low allele frequency of the variant in gnomAD, we were able to predict the damaging impact on the phenotype (Figure 2, Figures S1 and S2 and Table S3).

The p.Thr476Met (rs142763740) variant had a combined SSIP score of 0.69. In addition, DynaMut2 prediction showed a stabilizing effect of 0.32 kcal/mol with high confidence (Figure S1). The population frequency (gnomAD) of the variant (>0.0003) neither excluded nor confirmed the pathogenic effect. The variant was presented with an allelic frequency of 0.0038 in the Turkish Variome data. We therefore categorized the variant’s monoallelic phenotypic impact as low (Figure 2, Figure S2 and Table S3).

The p.Ile157Thr (rs17879961) variant is also expected to have a low pathogenic effect, with a combined SSIP score of 0.65, a population allele frequency of >0.003, and a confidence prediction of −1.22 kcal/mol (destabilizing effect). However, the results were quite variable when the SSIPs were analyzed further: seven SSIPs gave the lowest score for this variant. The findings regarding the p.Ile157Thr variant among the four identified variants imply that its monoallelic phenotypic impact is undetectable (Figure 2, Figure S2 and Table S3).

The p.Ser428Phe (rs137853011) variant scored the lowest among the four missense variants of interest, with an SSIP score of 0.62. In addition, a destabilizing effect of −0.37 kcal/mol was observed in the DynaMut2 stability prediction. Considering that the variant’s population allele frequency was >0.001, we concluded that the monoallelic phenotypic effect of this variant is also very low or unobservable (Figure 2, Figure S2 and Table S3). As a derivative approach, we recalculated the combined scores created from the dbNSFP ranked scores by weighting them according to 1-p-values (Table S3). Accordingly, the weighted SSIP scores (wISPPs) for p.Arg117Gly, p.Arg137Gln, p.Ile157Thr, p.Ile160Met, p.Glu351Asp, p.Ser428Phe, and p.Thr476Met were 0.50, 0.36, 0.28, and 0.30, respectively.

We also evaluated the pathogenic potential of p.Arg117Gly, p.Arg137Gln, p.Ile157Thr, p.Ile160Met, p.Glu351Asp, p.Ser428Phe, and p.Thr476Met based on the calculated ISPP scores (0.88, 0.59, 0.65, 0.63, 0.60, 0.62, and 0.69 respectively). Taking the allele frequencies in the study cohort, Turkish Variome, and gnomAD databases into account, we evaluated the pathogenic potential (Table 3, Table 4 and Table S3). Together with the ISPP scores and analysis of the allele frequencies, the increased frequency in the study cohort compared to the Turkish Variome and gnomAD data supported the pathogenic potential of these variants, especially p.Arg117Gly. Thus, these variants merit further investigation for their possible pathogenic role in *CHEK2*-associated phenotypes.

## 4. Discussion

Germline pathogenic variants in *CHEK2* have been reported to be associated with an increased risk of breast cancer, thyroid cancer, and kidney cancer and possibly inform colorectal cancer risk. However, recent wide cohort studies showed no increased risk of colorectal cancer in patients with *CHEK2* variations [8,20,21]. In this study, we aimed to determine the germline *CHEK2* variation frequency among Turkish cancer patients and individuals with cancer predisposition and evaluate the variants’ effects on CHK2 protein function using a new statistical approach. To the best of our knowledge, this is one of the first studies to investigate *CHEK2* variants in Türkiye and one of the most comprehensive in silico analyses of the *CHEK2* gene in the literature.

The *CHEK2* frequency in our study cohort was 8%. In the previous studies, White/European populations were over-represented, specifically the c.1100del variant. The frequency of P/LPs in *CHEK2* in the Carriers study, which included a large cohort of patients with breast cancer, was 1% [5]. The frequency of the c.1100del variation was reported to be 4.9–5.7% in Northern European breast cancer patients, where the frequency was 0.3–1.6% in unaffected British and Dutch populations [22]. In contrast, Southern European populations were shown to possess the same variation at very low frequencies [23,24]. In our study cohort, the frequency of the c.1100del variation was very low, and the variant was not detected in the Turkish Variome data [19]. The Turkish Variome database was created by combining more than 3000 exomes or genomes of non-cancer individuals with different neurologic, neurodegenerative, metabolic, or immunological disorders from Türkiye and is the only publicly available database representing the Turkish genome. A study from Greece also reported that c.1100del was rare in their breast cancer cohort, with a frequency of 0.16% [25]. This is not surprising since Greece and Türkiye share a gene pool due to their neighboring geographical locations. On the other hand, the most frequent *CHEK2* variation in our cohort was p.Thr476Met.

When we compared the variant allele frequencies in gnomAD with those in our study cohort, we found that almost half of the variations showed significantly higher allele frequencies than those reported in gnomAD. This would mean that some variants classified as VUSs in the gnomAD data could have been updated to LP using our cohort allele frequency by attributing the PS4 as moderate evidence. PS4 attribution can only be applied if the prevalence of a variant in affected individuals is significantly increased compared to controls [26]. In contrast, by applying the allele frequencies reported in the Turkish Variome database for available variations, we found that only three variations showed a significantly higher allele frequency in our study cohort. This finding highlights the importance of using population-specific allele frequency data during variant interpretation. According to our population data, the most well-known moderate/low-risk variant, p.Ile157Thr, was updated to VUS/LB. Physicians should be careful when they come across the following variants in cases of Turkish origin: p.Arg117Gly, p.Arg137Gln, p.Ile157Thr, p.Ile160Met, p.Glu351Asp, p.Ser428Phe, p.Thr476Met, and the splice variant c.592+3A>T. Boonen et al. reported an intermediate effect of p.Asp438Tyr and a damaging effect of p.Glu351Asp on the CHK2 protein [27]. These findings and the relatively high allele count in our study cohort suggest that these variants might damage CHK2, leading to a pathogenic phenotype. Notably, the variants p.Arg137Gln, p.Thr476Met, p.Ile160Met, and c.592+3A>T were not covered in the Boonen paper. While the current study did not find enough evidence to reclassify these variants as SSIPs, the significant increase in frequency of these variants among our patient cohort indicated that these variants may be considered P/LP in certain cases. Therefore, we posit that in Türkiye, when these variants are observed in patients and their families, expert review of pedigree is a necessity. If pedigrees exhibit concerning characteristics, these VUSs should be considered for management as P/LP despite insufficient robust data to classify them based on currently available evidence.

In a recent study, among a cohort of almost 40,000 hereditary cancer cases abundantly consisting of the White/European population, the most frequent monoallelic PV was c.1100del. p.Ile157Thr, p.Ser428Phe, and p.Thr476Met were excluded from the study due to the low incidence of BC among the carriers of these variants [8]. However, in silico tools predict these variants as P/LP. Today, various in silico tools have been developed, which differ in the evidence titles they use, and classification guidelines also use these tools in evidence attributions [9]. These tools incorporate evolutionary conservations, amino acid configurations, protein function, human variation data, and/or disease correlations. Most in silico prediction tools are trained for single-nucleotide variants (SNVs), which can create misleading data, especially for in/del and splice variants. We developed an in-house algorithm that evaluates 44 in silico prediction tools via a statistical approach and determines the most accurate gene-specific ISPPs [11]. According to our analysis, the p.Thr476Met, p.Ile157Thr, and p.Ser428Phe variants were not damaging enough to have any impact in monoallelic form. These variants were also reported as low-risk variants in hereditary cancer cohorts due to low correlations. Recently, the ACMG published a practice resource on managing *CHEK2* variants, in which the p.Ile157Thr variant is considered a low penetrance variant [7]. Although the monoallelic phenotypic effect of the p.Arg117Gly variation was determined as low, the combined SSIP score was higher than the other three low-risk variations. We hypothesize that the localization of p.Arg117Gly, central forkhead associated (FHA) domain, is essential for the localization of CHK2; indeed, it has been shown to contribute to sustaining lagging chromosomes [28]. Meanwhile, Boonen et al. showed the damaging effect of p.Arg117Gly by functional assay [27], which confirms our in silico prediction. Although p.Ile157Thr is also located in FHA, the amino acid (aa) change is milder than that of p.Arg117Gly. Arginine and glycine have been reported to be the most mutated amino acids [29]. Arginine is a positively charged, polar aa, whereas glycine is a small aa with high flexibility that can reside in regions where no other aa can be located [30]. Hence, the change from arginine to glycine severely affects the protein. Isoleucine and tyrosine are both hydrophobic aa, which may explain the difference between the effects of p.Arg117Gly and p.Ile157Thr. In line with our findings, Boonen et al. also reported p.Ile157Thr as functional [27]. It is also important to note that our in-house algorithm determined this difference between two variations in the same protein domain. This shows that using gene-specific in silico prediction tools is vital in variant interpretations during evidence attribution. Our interpretations based on in silico evidence and population data generally give results that align with the functional analyses. However, the two discrepant findings may have been due to the limitations of the Turkish Variome, a heterogeneous patient dataset from Türkiye.

This study presents one of the biggest cohorts from Türkiye for *CHEK2*. It is also the most comprehensive in silico analysis of *CHEK2* variations. The main limitation of this study was the unequal sex distribution. Since the most tested cancer type is breast cancer, the majority of the cohort was female, and males were not sufficiently represented. Although three major centers’ cohorts were analyzed, patients were mainly located in the Marmara Region of Türkiye, and we cannot claim that all ethnicities in the country were covered. Lastly, although very informative, comparison with the Turkish Variome dataset is limited since there are few cases with whole-exome sequencing in the database. Hence, we could not compare all *CHEK2* variants detected in the study cohort to a non-cancer Turkish population. The study cohort also presented concurrent variants in *CHEK2* gene. Our previous studies conducted on a White population showed a higher cancer risk in *ATM* + *CHEK2* or two *CHEK2* PVs than single gene variants [31]. Although the majority of the double variant carriers in this study were diagnosed with breast cancer, the number was limited to make a conclusion on genotype–phenotype correlations.

*CHEK2* is an important cancer gene, but it is hard for counselors or physicians to interpret when they encounter a VUS. Moreover, controversy exists in the *CHEK2* literature regarding variant classification and curation pertaining to whether the low-risk *CHEK2* variants (p.Thr476Met, p.Ile157Thr, and p.Ser428Phe) should be classified and managed as P/LPs. As emphasized by a recent editorial, there are no definitive guidelines on managing these common but low-risk variants in *CHEK2* [32]. Recent work has demonstrated that most missense P/LPs in *CHEK2* have a cancer-risk phenotype similar to loss-of-function mutations and are distinct from the three low-risk variants [8]. In this context, it is important to characterize and explore the prevalence of *CHEK2* variants among patients with cancer or hereditary cancer risk in Türkiye as these data will inform the counseling and care of patients or individuals with P/LPs vs. low-risk variants in *CHEK2*. Lastly, our in-house algorithm can be applied to other genes. It will aid in variant interpretation as we expand access to genetic testing across Türkiye for hereditary cancer risk and other genetic conditions. We hope our findings will inform care and counseling for patients and families identified as harboring *CHEK2* variants.

## 5. Conclusions

The variant allele frequencies in Türkiye differ from those found in Northern European populations. Three main *CHEK2* PVs (p.Thr476Met, p.Arg137Gln, and c.592+3A>T) were more highly present in the study cohort than the non-cancer group, and geneticists should carefully consider these while interpreting variations for patients of Turkish origin. Our newly developed statistical analysis on ISPPs could accurately predict the variants’ effects on the protein and highlights the importance of using gene-specific in silico tools during variant assessments. We believe that our findings will be informative for physicians and geneticists for the management of individuals with *CHEK2* PVs.

## Figures and Tables

**Table 1:** Characteristics of patients with *CHEK2* variations. Table summarizes the demographic and clinical characteristics of the 131 patients identified with *CHEK2* gene variants in the study.

Patient Characteristics	Number (%)
Number of Patients	131 (100)
Gender	
Female	126 (96.2)
Male	5 (3.8)
Mean Age * (min–max)	45 (19–70)
Diagnosis	
Breast Cancer	99 (75.57)
Unilateral	90 (68.7)
Bilateral	9 (6.87)
Colon Cancer	8 (6.1)
Tested due to Familial Risk	12 (9.16)
Multiple Primary Cancer	3 (2.29)
Other	9 (6.87)
Family History	
Yes	88 (61)
No	22 (16.8)
Unknown	21 (16.2)

* Mean age at the time of diagnosis.

**Table 2:** Summary of detected variants in the *CHEK2* gene. For each variant, the following details are presented: the serial number of the variants, the genomic position of the variant, the nucleotide change in the DNA sequence, the corresponding amino acid change in the protein sequence, the reference ID of the variant in the dbSNP database, and the classification of the variant according to the American College of Medical Genetics and Genomics (ACMG) guidelines. “P: Pathogenic”, “LP: likely pathogenic”, and “pathogenic/likely pathogenic” variants in ClinVar were assigned to the Pathogenic group; “benign”, “likely benign”, and “benign/likely benign” variants were assigned to the Benign group. Variants submitted under “VUS: variant of uncertain significance” were assigned to the Unknown group. fs*: frameshift variant creating a stop codon. If no amino acid change is applicable or no reference ID is available, it is marked as “-”.

Variant No	Genomic Location	Nucleotide Change	Amino Acid Change	dbSNP	ACMG Classification
V1	22:28694066	c.1427C>T	p.Thr476Met	rs142763740	Low risk-LP
V2	22:28725099	c.470T>C	p.Ile157Thr	rs17879961	Low risk-LP
V3	22:28709998	c.846+4_846+7del	-	rs764884641	LP
V4	22:28699929	c.917G>C	p.Gly306Ala	rs587780192	LP
V5	22:28719416	c.660_661delCA	p.Ile221Hisfs*23	-	LP
V6	22:28734432	c.290G>A	p.Trp97Ter	-	LP
V7	22:28695739	c.1100del	p.Thr367Metfs*15	rs555607708	P
V8	22:28695737	c.1232G>A	p.Trp411Ter	rs371418985	P
V9	22:28734664	c.58C>T	p.Gln20Ter	rs536907995	P
V10	22:28725070	c.499G>A	p.Gly167Arg	rs72552322	P
V11	22:28725278	c.409C>T	p.Arg137Ter	rs730881701	P
V12	22:28696924	c.1072C>T	p.Gln358Ter	rs878854909	P
V13	22:28703567	c.847-1G>A	-	rs878854926	P
V14	22:28711907	c.792+2T>A	-	-	P
V15	22:28694130	c.1376-13A>G	-	rs1064793330	VUS
V16	22:28725106	c.463T>C	p.Ser155Pro	rs1175278074	VUS
V17	22:28696929	c.1067C>T	p.Ser356Leu	rs121908703	VUS
V18	22:28695768	c.1201A>G	p.Thr401Ala	rs121908704	VUS
V19	22:28694072	c.1421G>A	p.Arg474His	rs121908706	VUS
V20	22:28695726	c.1243G>A	p.Val415Ile	rs1601721900	VUS
V21	22:28695190	c.1312G>T	p.Asp438Tyr	rs200050883	VUS
V22	22:28725277	c.410G>A	p.Arg137Gln	rs368570187	VUS
V23	22:28687968	c.1561C>T	p.Arg521Trp	rs533475838	VUS
V24	22:28725089	c.480A>G	p.Ile160Met	rs575910805	VUS
V25	22:28687973	c.1556G>T	p.Arg519Leu	rs587780180	VUS
V26	22:28734461	c.246_260delCCAAGAACCTGAGGA	p.Asp82_Glu86del	rs587780181	VUS
V27	22:28703507	c.906A>C	p.Glu302Asp	rs587780190	VUS
V28	22:28725118	c.451G>T	p.Gly151Cys	rs587781377	VUS
V29	22:28696943	c.1053G>T	p.Glu351Asp	rs587782268	VUS
V30	22:28703509	c.904G>A	p.Glu302Lys	rs587782460	VUS
V31	22:28689167	c.1510G>C	p.Glu504Gln	rs587782489	VUS
V32	22:28724974	c.592+3A>T	-	rs587782849	VUS
V33	22:28734552	c.170C>G	p.Ser57Cys	rs730881695	VUS
V34	22:28725020	c.549G>C	p.Leu183Phe	rs745646057	VUS
V35	22:28711965	c.736G>C	p.Val246Leu	rs748504161	VUS
V36	22:28687894	c.*2dupC	-	rs749257861	VUS
V37	22:28695743	c.1226A>T	p.Asp409Val	rs761095543	VUS
V38	22:28725031	c.538C>T	p.Arg180Cys	rs77130927	VUS
V39	22:28712015	c.686G>A	p.Gly229Asp	rs778212685	VUS
V40	22:28725265	c.1118A>G	p.Lys373Arg	rs786202446	VUS
V41	22:28725265	c.422A>C	p.Lys141Thr	rs786203192	VUS
V42	22:28703566	c.847C>T	p.Pro283Ser	rs1555917036	VUS
V43	22:28734441	c.281C>T	p.Ala94Val	-	VUS
V44	22:28725130	c.445-6T>A	-	-	VUS
V45	22:28695858	c.1110del	p.Thr367Mfs*15	-	VUS

**Table 3:** Comparison of altered allele counts and homozygous variants in the study cohort and gnomAD. Table compares the altered allele counts and the number of homozygous individuals between the study cohort (*n* = 3414) and the gnomAD database. The table includes statistical analyses to assess the significance of the differences between these two groups.

Variant No	Altered Allele Count (Study Cohort) (*n* = 3414)	Number of Homozygous (Study Cohort)	Total Allele Number (gnomAD)	Altered Allele Count (gnomAD)	Number of Homozygous (gnomAD)	Study Cohort vs. gnomAD *p*-Value	Adjusted *p*-Value gnomAD
V1	18	0	1,595,894	644	1	1.64 × 10^−14^	6.38 × 10^−13^
V2	8	0	1,614,104	4458	37	8.69 × 10^−1^	1.00
V3	4	1	1,428,834	41		4.44 × 10^−6^	1.73 × 10^−4^
V4	2	0	1,613,212	39	0	3.46 × 10^−3^	1.35 × 10^−1^
V5	1	0	NA	NA	NA	NA	NA
V6	2	0	NA	NA		NA	NA
V7	2	0	1,611,346	3467	5	4.08 × 10^−2^	1.00
V8	1	0	1,613,818	5	0	1.26 × 10^−2^	4.91 × 10^−1^
V9	1	0	1,613,966	102	0	1.96 × 10^−1^	1.00
V10	3	0	1,614,058	48	0	1.81 × 10^−4^	7.07 × 10^−3^
V11	1	0	1,614,006	32	0	6.74 × 10^−2^	1.00
V12	2	0	NA	NA	NA	NA	NA
V13	1	0	1,555,852	1	0	4.37 × 10^−3^	1.71 × 10^−1^
V14	1	0	NA	NA	NA	NA	NA
V15	1	0	1,556,040	16	0	3.66 × 10^−2^	1.00
V16	1	0	1,614,146	2	0	6.32 × 10^−3^	2.46 × 10^−1^
V17	5	0	1,612,796	7	0	3.28 × 10^−11^	1.28 × 10^−9^
V18	1	0	1,613,718	10	0	2.30 × 10^−2^	8.96 × 10^−1^
V19	1	0	1,596,022	73	0	1.46 × 10^−1^	1.00
V20	1	0	1,613,708	3	0	8.42 × 10^−3^	3.28 × 10^−1^
V21	13	0	1,613,498	606	3	1.38 × 10^−9^	5.37 × 10^−8^
V22	1	0	1,613,932	186	0	3.26 × 10^−1^	1.00
V23	1	0	1,595,990	72	0	1.44 × 10^−1^	1.00
V24	13	1	1,614,166	103	0	7.25 × 10^−19^	2.83 × 10^−17^
V25	3	0	1,595,820	205	0	1.04 × 10^−2^	4.05 × 10^−1^
V26	1	0	1,613,948	208	0	3.57 × 10^−1^	1.00
V27	1	0	1,437,854	14	1	3.49 × 10^−2^	1.00
V28	1	0	1,613,862	1	0	4.22 × 10^−3^	1.64 × 10^−1^
V29	4	0	1,613,522	105	0	9.25 × 10^−5^	3.61 × 10^−3^
V30	1	0	1,450,494	1	0	4.69 × 10^−3^	1.83 × 10^−1^
V31	1	0	1,595,222	23	0	5.00 × 10^−2^	1.00
V32	10	0	1,613,866	24	0	2.17 × 10^−19^	8.47 × 10^−18^
V33	1	0	1,613,974	2	0	6.32 × 10^−3^	2.46 × 10^−1^
V34	12	0	1,613,880	14	0	7.22 × 10^−26^	2.81 × 10^−24^
V35	1	0	1,613,088	3	0	8.42 × 10^−3^	3.28 × 10^−1^
V36	1	0	1,594,568	35	0	7.41 × 10^−2^	1.00
V37	1	0	1,613,664	2	0	6.32 × 10^−3^	2.46 × 10^−1^
V38	1	0	1,613,844	1337	0	5.40 × 10^−1^	1.00
V39	1	0	1,608,334	1	0	4.23 × 10^−3^	1.65 × 10^−1^
V40	1	0	1,613,500	2	0	6.32 × 10^−3^	2.47 × 10^−1^
V41	3	0	1,614,184	5	0	5.22 × 10^−7^	2.04 × 10^−5^
V42	1	0	1,554,814	1	0	4.38 × 10^−3^	1.71 × 10^−1^
V43	1	0	NA	NA	NA	NA	NA
V44	1	0	NA	NA	NA	NA	NA
V45	1	0	NA	NA	NA	NA	NA

**Table 4:** Comparison of altered allele counts and homozygous variants in the study cohort and Turkish Variome. Table compares the altered allele counts and the number of homozygous individuals between the study cohort (*n* = 3414) and the Turkish Variome database. The table provides statistical analyses to evaluate the significance of differences between these two populations.

Variant No	Altered Allele Count (Study Cohort) (*n* = 3414)	Number of Homozygous (Study Cohort)	Total Allele Count (Turkish Variome)	Altered Allele Count (Turkish Variome)	Number of Homozygous (Turkish Variome)	Study Cohort vs. Turkish Variome *p*-Value	Adjusted *p*-Value Turkish Variome
V1	18	0	5330	13	0	0.040855245	0.98052588
V2	8	0	7958	17	1	0.828720842	1
V3	4	1	NA	NA	NA	NA	NA
V4	2	0	7958	1	0	0.21624382	1
V5	1	0	NA	NA	NA	NA	NA
V6	2	0	NA	NA	NA	NA	NA
V7	2	0	7148	1	0	0.245875877	1
V8	1	0	7472	1	0	0.528893782	1
V9	1	0	NA	NA	NA	NA	NA
V10	3	0	7958	1	0	0.083823049	1
V11	1	0	7958	3	0	1	1
V12	2	0	NA	NA	NA	NA	NA
V13	1	0	NA	NA	NA	NA	NA
V14	1	0	7958	1	0	0.510313893	1
V15	1	0	5386	2	0	1	1
V16	1	0	NA	NA	NA	NA	NA
V17	5	0	7958	5	0	0.178647174	1
V18	1	0	7458	3	0	1	1
V19	1	0	NA	NA	NA	NA	NA
V20	1	0	NA	NA	NA	NA	NA
V21	13	0	7398	28	0	1	1
V22	1	0	7958	16	0	0.031830012	0.763920294
V23	1	0	NA	NA	NA	NA	NA
V24	13	1	7958	14	0	0.055944858	1
V25	3	0	5298	4	0	1	1
V26	1	0	7958	3	0	1	1
V27	1	0	7954	6	0	0.682301701	1
V28	1	0	7958	2	0	1	1
V29	4	0	7958	5	0	0.466282058	1
V30	1	0	NA	NA	NA	NA	NA
V31	1	0	NA	NA	NA	NA	NA
V32	10	0	7958	5	0	0.003652512	0.087660279
V33	1	0	NA	NA	NA	NA	NA
V34	12	0	NA	NA	NA	NA	NA
V35	1	0	7958	2	0	1	1
V36	1	0	5390	1	0	1	1
V37	1	0	NA	NA	NA	NA	NA
V38	1	0	7958	1	0	0.510313893	1
V39	1	0	NA	NA	NA	NA	NA
V40	1	0	7370	1	0	0.53295733	1
V41	3	0	NA	NA	NA	NA	NA
V42	1	0	NA	NA	NA	NA	NA
V43	1	0	NA	NA	NA	NA	NA
V44	1	0	NA	NA	NA	NA	NA
V45	1	0	NA	NA	NA	NA	NA

## Data Availability

The datasets generated during and/or analyzed during the current study are available from the corresponding author upon reasonable request.

## Supplementary Materials

The following supporting information can be downloaded at: https://www.mdpi.com/article/10.3390/cancers16223876/s1, Table S1: Concurrent variants detected in this study. Table S2: In silico pathogenicity predictor (ISPP) ranked scores from dbNSFP for all possible single-nucleotide substitutions in *CHEK2*. Table S3: Statistical calculation of the significant in silico predictors (SSIPs) and wSSIPs for the variants of interests. Table S4: *p*-values from the Kruskal–Wallis tests for the 44 ISPPs. Figure S1: Structural changes and conservation analysis of the CHK2 protein variants. (A) The changes in the bond structures of variants of the CHK2 protein. Newly formed bonds are indicated with arrows. The wildtype CHK2 at Arg117 has four clashes, one van der Waals, five hydrogen, five hydrophobic, and five polar bonds. The Arg117Gly variant changes the bond counts to one clash, two van der Waals, three hydrogen, zero hydrophobic, and three polar bonds, with a ΔΔG of −1.84 kcal/mol, classified as “destabilizing”. The wildtype CHK2 at I157 has zero van der Waals, four hydrophobic, and five polar bonds. The Ile157Thr variant changes these counts to two van der Waals, two hydrophobic, and six polar bonds, with a ΔΔG of −1.22 kcal/mol, classified as “destabilizing”. The wildtype CHK2 at Ser428 has four clashes, zero van der Waals, two hydrogen, zero hydrophobic, and four polar bonds. The Ser428Phe variant alters the bond structure to three clashes, three van der Waals, one hydrogen, six hydrophobic, and three polar bonds, resulting in a ΔΔG of −0.37 kcal/mol, classified as “destabilizing”. At Thr476, the wildtype CHK2 has four clashes, two van der Waals, six hydrogen, and 12 polar bonds. The Thr476Met variant modifies these to two clashes, one van der Waals, five hydrogen, and seven polar bonds, with a ΔΔG of 0.32 kcal/mol, classified as “stabilizing”. (B) The sequence logo represents the conservation of amino acids for each missense variant. Amino acid conservation across 12 species is shown above the x-axis, while structural conservation across five species is depicted below the x-axis. Despite varying degrees of amino acid conservation, structural conservation is observed at positions R117, I157, S428, and T476 of the CHK2 protein, with the 117th arginine showing 100% conservation across all analyzed species. Figure S2: Individual representation of the SSIPs ranked score comparisons. The violin plots represent the distribution of prediction scores for each variant, classified by clinical interpretation groups: B/LB (Benign/Likely Benign—blue), P/LP (Pathogenic/Likely Pathogenic—orange), and VUS (Variant of Uncertain Significance—green). Each violin plot includes a boxplot (centered line) showing the median and interquartile range for the scores within the group. The y-axis represents the scaled scores of the in silico prediction tools from dbNSFP, ranging from 0 to 1, where higher scores indicate a stronger likelihood of pathogenicity. Each variant is scored across multiple in silico predictors, with the clinical classification indicated by color. The violin shapes reflect the distribution of scores for each classification and predictor. File S1: Supplementary Methodology.
