# Supplementary figures and images for: Molecular and In Silico Analysis of the CHEK2 Gene in Individuals with High Risk of Cancer Predisposition from Türkiye
Source: Cancers (Basel). 2024 Nov 20;16(22):3876. doi: 10.3390/cancers16223876 (PMC11592704; doi:10.3390/cancers16223876)

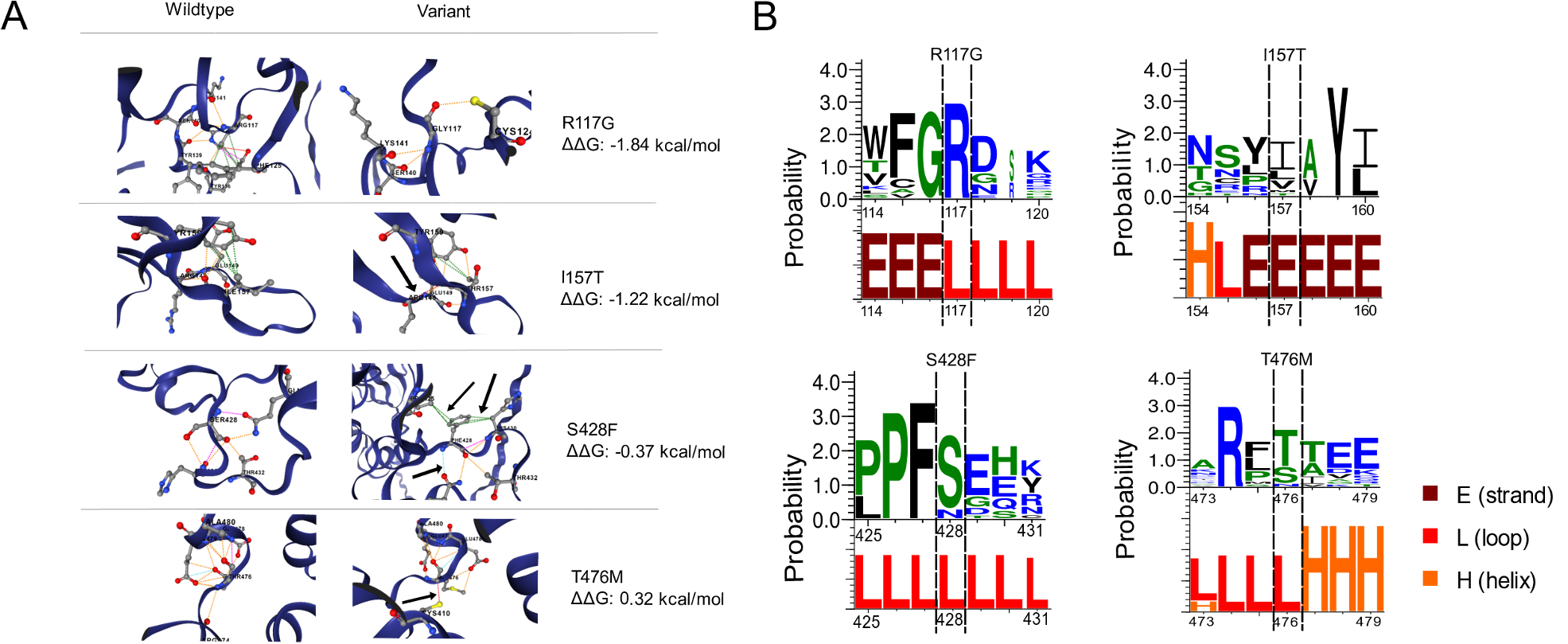

Supplement: Supplementary file 1 [file cancers-16-03876-s001.zip › Supp_FigureS1.png]

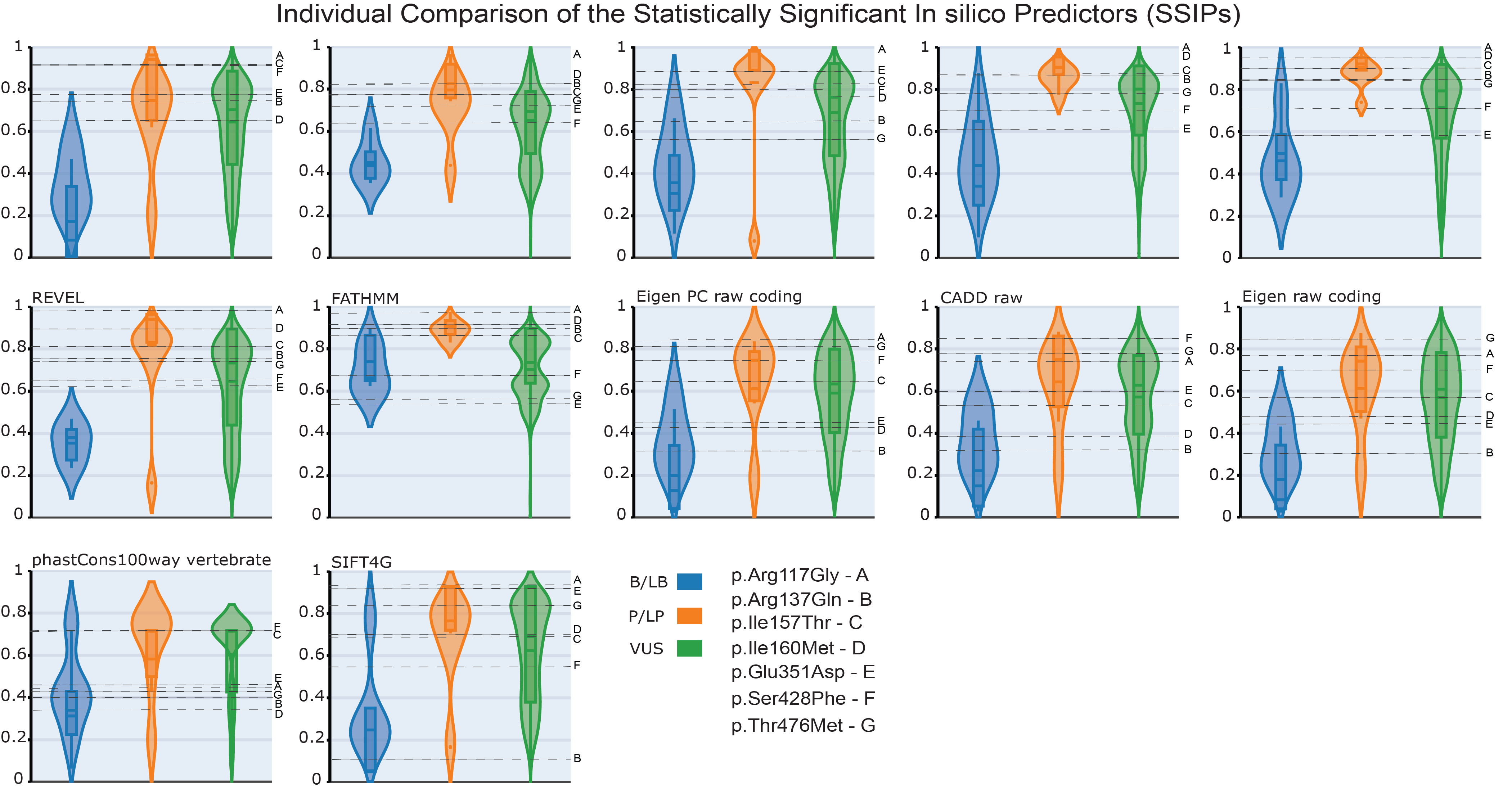

Supplement: Supplementary file 1 [file cancers-16-03876-s001.zip › Supp_FigureS2.png]
